# Adaptation of the Pharmacological Management of Delirium in ICU Patients in Iran: Introduction and Definition

**Published:** 2018-01

**Authors:** Mohammad Arbabi, Fatemeh Shahhatami, Mojtaba Mojtahedzadeh, Mostafa Mohammadi, Padideh Ghaeli

**Affiliations:** 1 Neuropsychiatry Section of Iranian Psychiatric Association, Roozbeh Hospital, Tehran University of Medical Sciences, Tehran, Iran.; 2 School of Pharmacy, Tehran University of Medical Sciences, Tehran, Iran.; 3 Department of Clinical Pharmacy, School of Pharmacy, Tehran University of Medical Sciences, Tehran, Iran.; 4 Imam Khomeini Hospital, Tehran University of Medical Sciences, Tehran, Iran.

**Keywords:** *Antipsychotics*, *Delirium*, *Intensive Care Unit*, *Pharmacological Treatment*

## Abstract

**Objective:** Delirium is a brain dysfunction syndrome. In most cases, this syndrome is neither diagnosed accurately nor treated properly. The incidence of delirium by itself increases hospitalization period, mortality rate and the cost in health spectrum. If appropriate attempts are not made to treat this complication, the outcomes could become worse. Thus, the present study aimed at conducting a review on medications which are prescribed to treat delirium and establishing a general view on their advantages and disadvantages.

**Method** **:** By searching Google Scholar, PsycINFO, Scopus, and PubMed databases as well as hand searching in key journals, data were collected without time and language limitation. After collecting the data, comparing the similar or contradictory information, and sorting them, the views of specialists were inquired and duly received via email. By acquiring consensus of opinions, the secondary manuscript was written in a narrative review form.

**Discussion:** This narrative review paper aimed at providing a general view on defining delirium, the pathologic factors that create it, and treating this syndrome based on its development. Authentic evidence regarding delirium management was reviewed and a treatment strategy was suggested for Iranian patients.

Delirium is a syndrome that is manifested by mental confusion due to perceptual and cognitive decline. There are many factors involved in its incidence, all of which lead to manifestation of a similar pattern of signs and indications in association with consciousness level and cognitive decline. Also, delirium has been known by other names including organic brain syndrome, ICU psychosis, and acute confusion state. 

Based on DSM-V criteria, delirium is an acute neuropsychiatric disorder characterized by a disturbed level of consciousness with reduced ability to focus, sustain, or shift attention, and it is accompanied by change in cognition, such as memory deficits, disorientation, speech and language disturbance, delusions, and perceptual abnormalities (1). 

The major characteristics of delirium include disorders in cognitive levels, attention, consciousness, function, and perception. Sleep disorder is another characteristic that relate to delirium. Sleep disorder, which is observed in delirium, includes drowsiness during the day, restlessness at night, and disorder in sleep continuation and/or disruption in sleep. In some cases, the sleep and awakening cycle is completely reversed (2). 

There is no need to observe the collective signs in patients to diagnose delirium. In fact, clinical symptoms of delirium differ as per the causes that create them. As an example, patients with bacteremia usually show encephalopathy and their delirium appears with a decline in consciousness (3). 

On the other hand, in patients with alcohol withdrawal syndrome due to the hyperactivity of sympathetic central nervous system, delirium is associated with common symptoms of restlessness, insomnia, chill, increase in heartrate, and hypertension (4, 5). 

In individuals with delirium, EEG usually appears in abnormal shape, and a general decline is usually observed in brain activities. Nevertheless, EEG usually shows accelerated activities in alcohol withdrawal syndrome, sedatives, and sleep pills deprivation syndrome. In addition, lab findings could be helpful in diagnosing delirium caused by poisoning or deprivation from a certain substance (6). 

In symptoms patterns, delirium is divided into 3 subtypes of hyperactive delirium, hypoactive delirium, and mixed delirium (7). Hyperactive delirium is mostly known by the symptoms of delusion and hallucination, confusion, agitation, stimulation, and restlessness. On the other hand, hypoactive delirium, involves sedation, lethargy, slow speech and confusion. Patients in mixed delirium state show features of both hyperactive and hypoactive delirium (ICU patients are usually not diagnosed with this subtype of delirium) (8). Diagnosing hypoactive and mixed delirium is usually more difficult. Hypoactive delirium, compared to its other types, mostly appears in elders and has the worst prognosis among other types of delirium (9). 

As an important and independent factor, delirium has many negative impacts on the clinical results of patients including increase in mortality rate, prolonging hospitalization, increase in treatment costs, functional disorders, and sustainability of long-term cognitive disorder with a dementia similar status (10-15). Nearly 60% of individuals who develop delirium suffer from subsequent permanent cognitive disorders; in addition, they are in risk of dementia 3 times more than other individuals (16-18). 

Hospital mortality in individuals with delirium ranges between6% to 8%, which is twice more than in individuals with no delirium records. In addition, the post-delirium mortality rate in individuals is 35% to 40% (18). In a prospective cohort study on 224 patients in ICU section, Ely et al, found that delirium caused a 3. 2 fold increase in the 6-month mortality rate and a 2 fold increase in hospitalization (11). 

Not only delirium incident, but also the duration of infliction is among the indexes that have negative effects on clinical consequences of patients (10). 

In addition, delirium is associated with increase in mechanical ventilation days and hospitalization in ICU, all of which lead to increase in health costs (11, 19, 20). Thus, presently, delirium has been recognized as a major health concern in ICU patients, imposing a high cost on both the patients and the health sector. 

However, unfortunately, despite its prevalence and importance, delirium is not taken seriously by physicians, and is not diagnosed in most cases, and most often it is managed poorly (7). Perhaps, this is due to the difficulty in diagnosis and absence of tools for easy measurement of delirium. Proper diagnosis and management of delirium by medical team is highly important, as the procedures they use are effective in the incident of delirium and its consequences (21-25). 

The aim of this narrative review paper was to provide a general view on defining delirium, the pathologic factors that create it, and treating this syndrome based on its development ground.

## Method

This narrative review was done by searching in Google Scholar, PsycINFO, Scopus, and PubMed databases as well as hand searching in key resource journals, and data were collected without time and language limitation. After collecting the data, comparing the similar or contradictory information, and sorting them, the views of specialists were inquired and duly received via email. By reaching consensus, the paper was written in the narrative review form. We aimed at collecting authentic evidence on delirium management and suggesting a treatment strategy for Iranian patients according to available medicines in Iran. 

**Pharmacological Therapy**

*Basic principles*

Pharmacological therapy is mostly used to control the symptoms of delirium rather than eliminating the primary cause of its development. Another point in treating delirium, along with efforts for understanding the main cause of its development, is attention to the age of the patient and the pathologic mechanism of delirium growth. The factor that develops delirium in the elderly is mostly related to illnesses, and the background problems are related to aging, which has different pathophysiological aspects than common factors, resulting in delirium in younger people (such as trauma, surgery). As a result, pharmacotherapy of delirium may differ when considering different factors resulting in the development of delirium including age. 

In patients older than 65, who are hospitalized in ICU, possibility of delirium incident increases 2% per year (26). This is due to problems and illnesses that appear as the individual grows older. As an example, most old patients suffer from associated diseases, such as diabetes, chronic heart disease, and arrhythmias and/or ischemic problems (caused by cardiovascular diseases or supraventricular arrhythmias), which might be symptomatic or not. On the other hand, aging is accompanied by age- associated changes in neurotransmitters of nervous system, decrease in cerebral blood flow and vascular density, loss of neurons especially in locus coeruleus, grey matter, and intercellular signal transmission systems (27-36). Vascular dementia is more noticeable in older patients than other patients. Dementia is the highest risk factor in delirium among older patients (13, 37 and 38). 

Another case, which causes delirium development in older patients, is aging-associated cholinergic inefficiency. Cholinergic system is one of the most important neurotransmitter adjustment systems in the brain, which controls memory-learning, cognition, and attention- associated activities (39, 40). For this purpose, one of the physiologic theories in the path of delirium is that delirium is caused by a disorder in the brain’s cholinergic transmission system (41, 42). Studies showed that acetylcholine decreases in the plasma and cerebral spinal fluid (CSF) of delirium patients (42-46). In addition, many studies have shown the relationship between medications with anticholinergic properties potential with delirium (27, 47-52). In fact, the serum anticholinergic activities level could serve as a delirium predictive factor (47), and by decrease in anticholinergic activities level, it will be possible to correct delirium (53). 

In addition, scientists have collected interesting data on some delirium patients, despite receiving no anti-cholinergic medication, have an increase in anti-cholinergic activities; hence, they assumed increase in age and/or some diseases could cause release of endogen substance with anti-cholinergic properties in body, which in turn plays a role in delirium etiology (45, 54). 

Dopamine is another neurotransmitter that plays a role in delirium. Dopamine level increases in some conditions, such as surgical operations, this increase in dopamine could give way to the emergence of agitation and delirium (55). Some believe delirium is a temporary psychological disorder caused by a decrease in cerebral cholinergic neurotransmitter in combination with an increase in dopamine transmission (27, 44). The cholinergic and dopaminergic systems interact via glutamate and γ-Aminobutyric acid (GABA) path (56). Some pharmacologic factors (opiates) and non-pharmacologic factors might cause delirium by increasing in dopamine and glutamate activities simultaneously with acetylcholine in access (57). 

One of the reasons that makes older people more vulnerable to delirium is inflammation phenomenon; moreover, age-associated changes in immune system and increase in cytokines excretion by adipose tissue cause a chronic inflammation in older patients (58). This inflammation might be involved in the advancement of diseases via inflammatory mediators generation. The aging process is associated with 2 to 4 times increase in the inflammatory mediators base level including cytokines and acute phase proteins (59, 60). On the other hand, central system response to inflammatory mediators might increase in patients at risk delirium, such as old patient or individuals with central neurology system diseases. It seems that the number of microglia cells that have the duty of protecting nervous system increase in those patients; in turn, those cells are prepared to provide a fast and intensive response to stress generating factors (61). 

Other factors that might increase the risk of delirium in elderly are decrease in cognitive capacity, lower metabolic capacity, hypersensitivity, allergies to medications, and reduction in tolerance of anti-cholinergic effects of medications (62). 

Pharmacological treatment should not be an initial step to treat delirium in elderly unless narcotics are needed for pain control to prevent dangerous consequences of pain. Moreover, the pharmacological approach is saved for when the individual is intensively agitated (hyperactive delirium subtype). It is important to note that many cases of delirium in elderly are due to ischemia, low perfusion, or low cholinergic activity. Since antipsychotics (e. g. haloperidol) demonstrate anticholinergic properties, these medications can intensify arrhythmias and subsequent ischemic problems and should be avoided to control delirium as much as possible. 

In general, when deciding to prescribe medications for the management of delirium, one must note whether or not vascular dementia, regardless of age, is an issue. Although heart infarction and brain stroke are more common in old patients, nowadays, most cases of heart infarction and brain stroke occur in individuals younger than 65. In case of vascular dementia, medications with anticholinergic properties, such as haloperidol, could intensify delirium and/or even cause its development. 

However, there is another group of patients in whom delirium is not caused by cholinergic deficiency or ischemic phenomena. Most of these patients are young patients, whose delirium is the result of pain and problems caused by damage associated molecular pattern (DAMP) and/or activation of neurohormonal systems related to adrenergic system. 

One of the reasons that could cause delirium includes processes such as surgery, trauma, and/or infections that would lead to acute inflammation. This state leads to the activation of brain parenchyma cells, pre-inflammatory cytokines, and inflammatory mediators in central nervous system cells, which in turn causes neurological and synapse disorders, followed by disorders in neurological and cognitive activities, also known as delirium (63, 64). Factors that stimulate immunity systems, such as pathogen associated molecular pattern (PAMP) that originate from micro-organisms or DAMP molecules inside the body, transmit signals to the brain from the neurons and hormone paths and cause pre-inflammatory cytokines production and excretion by macrophages cells. Several studies have shown that amount of C- reaction proteins, interleukin-6 (IL-6), tumor necrosis factor alpha (TNFα), interleukin -1 receptor antagonist (IL-IRA), interleukin-8 and interleukin-10 in delirium patients increases compared to other patients (65-67). 

Some physiologic processes, such as tissue damages, hypoxia, acute diseases, and various infections, are accompanied by an increase in energy demand and reduction in brain oxidative metabolism. In some cases this oxidative stress, and deficiency in antioxidants could lead to brain disorders including delirium (68-71). 

Delirium could develop in reaction to acute stress conditions. Glucocorticoids level goes up in stress conditions; and thus, the ability of neurons in saving them from various metabolic conditions decreases and a general pathology is created in brain neurons (72). 

To control delirium and improve the symptoms, it is possible to use short-effect combination of haloperidol and benzodiazepines. Even opiate prescriptions for pain control cause less risk of delirium intensity. Note that in this group, vessels are healthy and there is no ischemia or vascular dementia; rather, the activities of neurohormonal systems significantly increase. 

The third group consists of substance or alcohol abusing patients in whom, delirium appears as a result of giving up those substances (73). These patients usually show hyperactive delirium. This type of delirium can occur even upon sudden deprivation from sedative and/or opioid drugs, which are usually prescribed for long term, as a part of a routine pharmacologic therapy process in controlling pain in ICU wards (74). 

In DSM-5, withdrawal delirium or delirium tremens (DT) is defined as, the case when an individual, in addition to diagnostic criteria for delirium, is subject to alcohol deprivation criteria as well, which includes the following (1):

- Stop or decrease in excessive use and/or long-term use of alcohol

- At least 2 cases of the 8 symptoms might appear after reducing alcohol consumption. The symptoms include autonomic system hyperactivity, shaky hands, insomnia, nausea, vomiting, delusion, and passing hallucination, psychomotor confusion, and generalized tonic-clonic seizure. 

Ethanol addiction is observed in 15% to 20% of hospitalized patients (75). Around 8% to 31% of hospitalized patients with alcohol addiction, especially those with history of surgery or trauma and exposure to alcohol withdrawal syndrome (AWS), may show neurologic and autonomic signs. Amino-acid neurotransmitters play a dominant role in the pathophysiology of alcohol deprivation. Constant consumption of alcohol leads to reduction in the gamma-aminobutyric acid receptors performance and increase in N-Methyl-D-aspartic acid (NMDA) receptors. Both those mechanisms can put the patient at risk of alcohol withdrawal syndrome (2, 76). The spectrum of AWS symptoms could manifest mild to terminal symptoms (77). More than 15% of hospitalized AWS patients experience generalized tonic-colonic seizure, and 5% show delirium tremens. In fact, AWS is a life risking combination of neural system stimuli (agitation, delirium, seizure) and hyperadrenergic symptoms (hypertension, tachycardia and arrhythmia) (78). ICU patients with intensive alcohol deprivation have more dependence to ventilator system and longer term of ICU hospitalization caused by the continuation of their delirium (78-80). In delirium caused by alcohol deprivation, one must note 2 points; first, previous addiction to ethanol is usually ignored in ICU care which puts patients in AWS and DT risks; second, at AWS and DT measurement tools have not been validated in ICU so far due to which, distinguishing delirium caused by alcohol deprivation from other cases could be difficult. 

The main goal in treating these patients is to control their anxiety and agitation, lowering the risk of seizure, damages and death. For this purpose, pharmacologic therapy with haloperidol plus benzodiazepine and narcotics must be used for this group of patients. Unlike senile patients, haloperidol medication in this group of patients decreases mortality and plays a major role in treatment. 

**Antipsychotics**

One of the theories of delirium development is the increase in dopamine excretion. Some believe delirium is a temporary disorder caused by reduction in cerebral cholinergic neurotransmitter in combination with an increase in dopamine transmission (27, 44). Some pharmacologic factors (opiates) and nonpharmacological approaches (surgery) might cause delirium through an increase in dopamine and glutamate activities and acetylcholine decrease in access at the same time (57). It was based on this hypothesis that antipsychotics were introduced in treating delirium (81). 

Haloperidol, a dopamine blocker antipsychotic, has been the most frequently prescribed medication to treat delirium. A study was conducted on 989 ICU patients who had undergone mechanical ventilation for more than 48 hours. Among individuals who participated in the study, 83 received haloperidol medication. Patients in haloperidol group received a mean daily dose of 11. 5mg haloperidol for 3. 5 days. The study compared differences in hospital mortality between patients who received haloperidol within 2 days of initiation of mechanical ventilation and those who never received haloperidol. The results of that study showed the treatment with haloperidol during mechanical ventilation was associated with significantly lower hospital mortality for mechanically ventilated ICU patients (82). 

There are other controlled studies that show taking antipsychotic medications could be effective in treating agitation in patients (83, 84). Nevertheless, delirium is not diagnosed by standard measurement tools; rather, solely other symptoms including confusion and lack of recognizing time and place have been taken as the evaluation criteria in most studies. Although haloperidol is usually prescribed in treating delirium, one must consider that, to date, no valid and strong data have been published to prove the safety and efficiency of prescribing it. The recent guideline of the American Association of Critical Care which was released in 2013 (8), addresses this point through such phrases as “there is no proven evidence to show delirium treatment with haloperidol would reduce delirium sustainment in ICU adult patients.”, or “There has been no perspective clinical trial in the past decade about the safety and effectiveness of using haloperidol to treat delirium, specifically, in hospitalized adult patients.” Most studies, in which haloperidol was used in the treatment of delirium, have reported a decrease in confusion following haloperidol administration. Haloperidol consumption has also shown a decrease in the use of narcotics or sedatives. In any event, confusion, a morbidity factor in delirious patients, was decrease after haloperidol consumption (85) and this is one good reason to prescribe this medication for the treatment of delirium. 

Recently, there have been some studies on using atypical antipsychotics in treating delirium (86-88). In a clinical trial on 73 patients in ICU section, oral haloperidol was compared with olanzapine in treating delirium. No difference was observed between the 2 groups; nevertheless, 13% of patients who received haloperidol suffered from extrapyramidal symptoms, while none of the patients who received olanzapine manifested that symptom. Among restrictions of this study, are insufficient randomization design of the study, small sample size, and lack of blindness of the physicians and nurses. In addition, there was no placebo recipient group in the study (89). 

In another clinical trial, typical and atypical antipsychotics were studied in 101 patients under mechanical ventilation. No difference was found in the number of delirium or coma days in patients who received haloperidol, ziprasidone or placebo. Furthermore, with respect to extrapyramidal symptoms or side effects, there were no significant differences among the number of mechanical ventilation days, number of ICU admission days and/or mortality rate among the 3 groups (90, 91). In a Cochrane type review study, it was revealed that typical antipsychotics when used for a short time (e.g., treating delirium) were as efficient as atypical antipsychotics. In addition, they showed to be safer than atypical antipsychotics (92). 

However, there is still a controversy on priority of using typical antipsychotic medications (such as haloperidol) or atypical (such as aripiprazole, olanzapine, quetiapine, risperidone, ziprasidone in treating delirium). Using atypical antipsychotics does not appear to have much of efficiency in treating delirium in ICU patients, as in those patients, delirium is an acute incident which has developed temporarily in the patients as a response to pathologic conditions. Therefore, treatment must be provided in the shortest possible time to prevent negative consequences. There are 2 points that must be considered in atypical antipsychotics. First, injectable atypical antipsychotic forms are not available in Iran and oral forms of these medications show their effects with delay, which is not a desirable when treating delirium. Second, all studies on atypical antipsychotics have reported a significant decrease in delirium symptoms after prescribing these medications. However, in none of those studies, the control and placebo groups were used to blind the study. One must take this issue into account that delirium is a syndrome with fluctuating symptoms in its nature and sometimes the patient recovers with no intervention; besides, delirium disappears when its cause is removed. Therefore, due to the lack of strength in the design of those studies (including absence of placebo group), researchers cannot be certain whether or not the improvement and decrease in delirium symptoms is due to atypical medications prescription, or the credit goes to the elimination of the development ground. 

In treating delirium by typical antipsychotics, butyrophenones are preferred to phenothiazines since phenothiazines (such as chlorpromazine, phenothiazine, thioridazine, trifluoperazine, and promethazine) are associated with those adverse effects such as drowsiness, anticholinergic effects, and α-adrenergic blocker effects that might worsen delirium. Butyrophenone, especially haloperidol, is used as the safest and most effective antipsychotic medication in treating delirium. Haloperidol is a high potent dopamine blocking factor and has less anticholinergic adverse effects than other medications in this class. In addition, haloperidol, with minimum cardiovascular adverse effects and lack of active metabolite, is generally considered the first line medication in delirium treatment among antipsychotic medications. 

There are reports on QT prolongation with haloperidol in delirium treatment. Increase in QT could cause Torsade de point arrhythmia. Torsade de point arrhythmia is a dangerous symptom, which is related to with antipsychotic medication consumption and could lead to ventricular fibrillation and sudden death. There are several reports on the incidence of this arrhythmia following IV haloperidol injection (93-96). This incidence can occur in ECG without increase in QT distance (97, 98). However, the frequency of this serious symptom is related to high dosage of IV haloperidol (>35 mg/day); nonetheless, it has also been observed in lower dosage of haloperidol in IV or oral form as well (94, 99). In general, antipsychotics are not recommended for patients with specific risk factors for Torsade de point arrhythmia such as patients with increased QT, patients who take other medications at the same time, increasing their QT or patients with history of arrhythmia. Therefore, it is wise to have ECG report before prescribing antipsychotic medications and monitor QT-interval. Electrocardiography is required in old patients. Increase in the wave distance between QT to 450 msec or more, or 25% increase in QT distance or more compared to previous ECG of the patient, requires seeking a cardiologist’s advice to start haloperidol medication therapy; and if this occurs in the course of the treatment, there should be a decrease in dosage, or the prescription of the medication should be stopped (93-95). In addition, it is recommended to monitor magnesium, potassium, and phosphorus levels in patients, especially in patients whose QT wave distance is equal or more than 440 msec, or in patients who take other medications at the same time with the side effects of QT prolongation, or patients who suffer from electrolyte disorders (100). It is because of this serious symptom that haloperidol must not be prescribed in old patients with ischemic risk until the cause of delirium development in them is found. Due to their arrhythmogenic characteristics, antipsychotic medications intensify ischemia and complications related to perfusion. 

Consumption of antipsychotics could be associated with extrapyramidal symptoms accompanied by neurological symptoms, motor disorders, and neuroleptic malignant syndrome (NMS). There have been reports on NMS symptoms with haloperidol in several case reports. Patients with brain traumatic damages show more sensitivity towards this symptom (101). The extrapyramidal side effects mostly occur in high dosage of typical antipsychotic medications. Those symptoms include akathisia, acute dystonic reaction (more often in young male patients), parkinsonism (more in old females), and tardive dyskinesia (in patients who take first generation of antipsychotic for long period) (102). 

Lowered seizure threshold, galactorrhea, and increase in hepatic enzymes and motor disorders are among adverse effects of antipsychotic medications. Haloperidol is contraindicated for patients with parkinsonism and dementia with lewy bodies. 

The starting dosage for delirium treatment is 2 to 10 mg depending on age, weight, and severity of symptoms. In case of no sufficient response, the dosage could be increased to double the initial dosage every 15 to 30 minutes until the patient is calmed. When the patients’ symptoms are controlled, 25% of the last dosage is repeated every 6 hours and the dosage is lowered in this order. For old patients with no ischemic and perfusion issue haloperidol must be started with lower dosage by considering pathologic factor which contain no risk for the patient. For example, 0. 25 to 0. 5 mg haloperidol is prescribed each 4hours. On the other hand, patients with more intensified agitation disorders might need higher dosage. The general effective dosage for most delirium patients is 5 to 40 mg. Higher dosage of haloperidol to 1200 mg/day and >200 mg/day for 15 days has been already reported (103, 104). Information on safety in using high dosage of typical antipsychotics is obtained only from a few case reports available (105, 106). 

When prescribing haloperidol in ICU patients, intravenous injection is the fastest and most effective way in the treatment of delirium. On the other hand, IV injection of medication causes less extrapyramidal effects (107). Haloperidol IV prescription has not received FDA approval for delirium treatment. One study on the effects of continuous infusion of haloperidol in patients who needed several doses, indicated that this form of prescription has a safer profile since it does not even create problems like lowering blood pressure even when used in several doses (106). Therefore, by considering the results of this study, it is recommended to prescribe haloperidol infusion in patients who need more than 8 doses of 10mg haloperidol in 24 hours, or more than 10 mg per hour in 5 subsequent hours. 

In addition, due to adverse effects that might appear in haloperidol injection, some additional terms must be included in the prescription to hold the injection; for example, in case of sodium fall to less than 136 Eq/L due to water and salt retention caused by haloperidol, upon noticing premature ventricular contraction (PVC) and premature atria contraction (PAC) in the patient’s heartbeat and semi-seizure motion; thus, it is better to stop injection until the patient’s condition is stable. 

**Benzodiazepines**

There are few studies on using benzodiazepine as monotherapy in delirium management, and that limited information reveals single therapy by benzodiazepines is not generally recommended for delirium treatment. Due to their CNS depression effects and sedation, benzodiazepines could be one of the risk factors in delirium development, except in delirium for giving up benzodiazepine and alcohol (20, 26 and 108). Although currently the relationship between using benzodiazepines and risk of delirium development is debated (109), especially due to undesirable adverse effects of antipsychotics, there is an increasing trend to use them when antipsychotics are contraindicated. 

On the other hand, lack of sleep is another risk factor in delirium and benzodiazepines are used in ICU for their sedative and hypnotic effects. Aziawa et al studied the use of medications for improving post-surgical sleep in patients, who had undergone intestine operation. In that randomized, prospective and small-scale study, one group received IM injection of diazepam every night and an IV flunitrazepam and pethidine, infusion every 8 hours after operation for 3 nights, and the control group received no sedative or sleeping pills. Of the 20 patients, 7 in the control group (35%) and 1 in benzodiazepine and pain killer recipient group (5%) developed delirium. The authors concluded that disorder in sleep and awakening cycle was one of the most important factors that result in post-surgical delirium. Therefore, using sedatives to improve sleep and control sleep-awakening rhythm might play a role in preventing delirium (110). 

There are records on mixed use of benzodiazepines and antipsychotics in treating delirium, the results of which show this combination, in addition to lowering side effects, could increase clinical effectiveness in a specific population consisting of critically ill cancer patients or those with AIDS. The results of several studies on using haloperidol in IV form with IV lorazepam indicated that a combined treatment is a more effective method along with lowering delirium period and less extrapyramidal than treatment with haloperidol alone (111-113). 

Using benzodiazepines is not recommended as a single treatment in delirium. However, in alcohol and benzodiazepines withdrawal delirium (76, 114), in delirium caused by unknown narcotics, delirium caused by hallucination, cocaine and stimulators, benzodiazepines can be useful as monotherapy. Benzodiazepines have been known as the major treatment in treating alcoholism; however, their safety and efficiency have not yet been fully (10, 115). 

In addition, there have been cases when benzodiazepines replace antipsychotics as the first line of treatment. For example, when we need a medication to elevate seizure threshold unlike antipsychotics, benzodiazepines reduce seizure threshold. Even in delirium due to seizure, benzodiazepines are the first choice of treatment. In addition, when anticholinergic or akathisia caused by antipsychotic medications worsens the patient’s condition, benzodiazepine monotherapy is used in treating delirium. Since haloperidol use is contraindicated in patients with Parkinson and lewy body dementia, benzodiazepines are used as the first line in treating delirium in these patients. Using antipsychotics are more dangerous in elderly and should not be used as first line treatment in this population. In old patients with delirium, benzodiazepines are in first line of treatments; however, one must note that benzodiazepine might lead to respiratory depression especially in older patients with lung problems. 

Benzodiazepines are accompanied by side effects such as sedation, aggressive behavior, amnesia, akathisia, respiratory depression, physical attachment, recurring insomnia and delirium. Older ones are at higher risk to suffer those complications (112, 116). In hepatic encephalopathy, benzodiazepines should not be prescribed since they cause accumulation of glutamine which is chemically a byproduct of gamma- aminobutyric acid (GABA). 

In prescribing benzodiazepine, only one with a short half-life and without any active metabolite like lorazepam should be used. Midazolam also has a short half-life, is metabolized via CY3A4 enzyme and is metabolized to the active metabolite, 1-hydroxyl methyl midazolam. Since lorazepam is not available in injective form in Iran, midazolam could be a suitable alternative. 

There are few studies on optimum dosage of benzodiazepine in delirium treatment. For midazolam, the starting dose could be 1to5 mg and it can be repeated every 15 to 30 minutes. In the delirium caused by alcohol or sedatives withdrawal syndrome, higher dose of benzodiazepines is necessary. There are reports on high dose of midazolam even up to 2850 mg in 5 days without any respiratory adverse effects (117). 

**Melatonin**

Melatonin is one of the medications currently receiving special attention for its properties in preventing and treating delirium. This is because of the role of this substance in regulating sleep-wake cycle (118). There has been always a belief that disorder in sleep-awakening serves as a pathologic factor in developing delirium (119). It was for the same reason that a Scottish surgeon in 19th century called delirium as the diseased dreams (120). Melatonin is a hormone excreted from pineal gland and plays an important role in regulating sleep-awakening cycle, and disorder in its excretion could be a factor in developing delirium (119) and psychosis (121) due to disorder in sleeping-awakening cycle. 

Benzodiazepine could be used to regulate sleeping; however, the difference between melatonin and benzodiazepines in sleep is that benzodiazepines and other hypotonic medications could increase sleeping time in general, but they cause interruptions in natural phases of sleep (122). 

Disorder in sleep and awakening cycle is a common incident in hospital wards, which is caused by factors such as pain, side effects of medications, noise, and unfamiliar surrounding environments. In addition, sleep disorder in ICU patients and loss of day-night rhythm could be due to metabolic, immunity, neurologic and respiratory disorders, as well as mechanical ventilation (122, 123). Studies show ICU patients, who experience sleep disorder, are more in risk of developing delirium than patients who do not have such problems (124, 125) Therefore, the quantity, and more importantly, the quality of sleep, particularly in ICU old patients who are more vulnerable to develop delirium, have been considered as one of the goals in preventing delirium. Sleep disorder does not only affect ICU patients; for example, studies on patients who underwent heart surgery showed that this group of patients developed delirium more often due to their constant sleep deprivation (126, 127). 

On the other hand, studies on prescribing melatonin in ICU patients showed improvement in quality of sleep and its longer period (128) In addition, data have shown prescribing melatonin in low dose, melatonin exogenous as a preventive measure, could decrease delirium development (129, 130) and/or reduce the intensity of agitated behaviors in old patients (131). 

A case report was published by Hanania and Kitain in 2002 on preventive effects of melatonin in delirium in a 78-year old male with post-surgical delirium history (132). When the patient was hospitalized again for another surgery, he received melatonin for 3 nights after surgery. Following this surgery, he did not show delirium symptoms. The report writers concluded that melatonin prescription in period could be a suitable alternative, as melatonin level trends to decline in such conditions. 

In another case report, each of the 2 presented patients consumed 2mg melatonin 3 to 4 nights after the surgery, as treatment for one patient and preventive measure for the other one. It was noted that the medication proved effective in reducing delirium without creating any side effects (132). 

The plasma level of melatonin could be related to delirium. Several studies have shown a relationship between abnormal plasma level of melatonin in day-night rhythm and post-surgical delirium (123, 133). A study was conducted on older patients in a hospital. In that study, the mental status of the patients was constantly evaluated by dementia rating scale, and at the same time, their urine concentration of 6-sulfamethoxy melatonin was also evaluated as the main melatonin metabolite in the body. Based on the date of the study, the authors concluded that reduction in melatonin level is associated with delirium (134). In another study, they found that in the amount of 6-sulfamethoxy melatonin decreased in hyperactive delirium and it increased in hypoactive delirium (135). 

Another study in adult patients in post-surgery delirium showed that melatonin level in those individuals decreased 2 days after the surgical procedures compared with the pre-operation level (136). In addition, this study showed patients with delirium with other factors including infections had higher levels of melatonin. Shigeta measured melatonin excretion in patients who had undergone abdominal surgery (137). The plasma level of melatonin was measured once per 2 hours, 1 day before and 1 day after surgery in 19 delirium-free patients and 10 patients with delirium. The results showed that by increase in age, especially in patients over 80 years of age, there was a general reduction in melatonin excretion in the individual. The least amount of melatonin excretion occurred in an old patient, who underwent surgical operation. Therefore, there are changes in the quantity and rhythm in melatonin, which are related to delirium. In addition to the role of melatonin in sleep regulation, this substance plays an important role in several physical functions, the very fact that creates its potential in developing delirium. 

In addition to the chronobiotic effect (that is, effects on different aspects of biotechnological structure in bio clock of the body) and regulating sleep- awakening cycle, melatonin can have a role in recovery of the symptoms in the 24-hour rhythm, and trapping free radicals with its high antioxidants property. In addition, melatonin has anti-inflammatory and analgesic characteristics (138-140). 

Owing to these characteristics, melatonin could create a natural protection to learning and memory mechanisms (130, 138-143). 

Nevertheless, there is no study with large-scale statistical population and strong plans on melatonin cases to serve as a tool and source of data collection on melatonin prescription in post-surgical patients and ICU patients in a routine form, which could be employed in providing recommendation for such attempts. However, as mentioned above, the studies that have already been performed all aimed at determining usefulness of melatonin in delirium improvement. On the other hand, melatonin is a medication with low side effects; hence, it could be recommended for patients, whose delirium is assumed to be caused by sleep disorders. 

**Alpha 2- Central Agonist**

Dexmedetomidine is a selective Alpha 2-adrenergic agonist with no effects on GABA receptors and is used in ICU as a sedative and sleep medication. Dexmedetomidine has relaxing effects, reduces anxiety through effects in locus ceruleus receptors, has analgesic property by spinal cord receptors, and reduces the effects in response to stress without respiratory depression (144, 145). In addition, it has been said this medication provides more normal sleep rhythm than benzodiazepine (145-148). A meta-analysis of clinical trial on ICU patients following elective heart surgery showed that dexmedetomidine reduces the duration of ICU hospitalization in a moderate form (143). 

Studies have been performed so far on comparing benzodiazepines and dexmedetomidine and their effects in manifestation of delirium. A study showed that dexmedetomidine can have special effects in reducing the risk of delirium in comparison with benzodiazepines in patients under mechanical ventilation. In this controlled clinical trial performed on 106 patients under mechanical ventilation, it was found that compared to lorazepam, dexmedetomidine led to considerable decrease in delirium or coma free days (23). In a randomized open label clinical trial, which was performed on the effects of sedative medications on post heart surgery patients, the researchers concluded that dexmedetomidine causes less risk of delirium compared to other sedative medications (149). The amount of delirium incidents that used dexmedetomidine as a sedative medication was 8% and in a group, who used midazolam or propofol, the incident was 50%. In general, studies that compared sedation between benzodiazepines and dexmedetomidine reported that the duration of delirium in patients who received dexmedetomidine was significantly lower than the group who received benzodiazepine (90, 150 and 151). This information is not comprehensive on whether or not benzodiazepine could increase the risk of delirium, or if dexmedetomidine could reduce this risk; thus, judging on this matter needs more researches. These findings led to the recommendation that using dexmedetomidine as sedation should be a priority in patients, except those patients who have developed delirium as a result of ethanol consumption or sudden cut in benzodiazepines. 

In addition to positive effects of dexmedetomidine to other medications in lowering the incident of delirium, several studies found other advantages and disadvantages for this medication in treating delirium, which must be considered in selecting them for treating delirium. Jacob et al. published 2 clinical trials on comparing dexmedetomidine with midazolam and dexmedetomidine with propofol. They found those 3medications have are not different in their effects on hospitalization period in ICU; however, those groups that received dexmedetomidine could bear pain better. In addition, dexmedetomidine lowers the duration of mechanical ventilation compared with midazolam; however, they did not have any differences with propofol. Dexmedetomidine mostly causes bradycardia and hypotension in comparison with midazolam and in comparison with propofol, they result in more first class atrioventricular block (152). In 2007, a study was conducted on comparing midazolam and dexmedetomidine in sedative characteristics in critical patients in acute status hospitalized in ICU (150). The first hypothesis in the study claimed that dexmedetomidine would improve clinical outcomes in patients versus GABA agonists, such as midazolam. Moreover, and it was observed that frequency of delirium incident in dexmedetomidine recipient group was 54% (132 patients out of 244), while in midazolam recipient group, the frequency of incident was 76. 6% (93 patients out of 122). Despite the similarities of the 2medications in sedation, there were several important differences which were expressed in this prospective, double blind and random study. In dexmedetomidine recipient group, bradycardia was more frequent, while hypertension and tachycardia were more common in the midazolam recipient group. There was 20% less delirium report among patients who received dexmedetomidine than midazolam group; on the other hand, the midazolam group was separated from ventilator at least 2 days earlier. 

There have been reports on bradycardia followed by cardiac arrest in patients who received dexmedetomidine (153, 154); thus, in patients with heart problems or patients who might be at risk of serious bradycardia in case of receiving dexmedetomidine, this medication must be prescribed with caution. The patient’s heartbeat and blood pressure should be monitored as well when this medication is prescribed. 

In a study dexmedetomidine was compared with haloperidol in 20 ICU patients who showed agitation caused by delirium, and it was found that ICU hospitalization period of dexmedetomidine recipients was shorter than that of the other group. However, lack of blinding of the study and small sample size of the study were among the restrictions in that study (155). 

As mentioned above, dexmedetomidine does not replace benzodiazepines as the first line treatment delirium caused by alcohol withdrawal, or so called delirium tremens; however, in patients who do not respond to high dose of benzodiazepines, especially those who are under mechanical ventilation, dexmedetomidine in 0. 7mg dosage per hour can be used as the second line of treatment (156, 157). 

Clonidine is another medication in this class that is available in Iran. This medication could be used in oral form in low dosage in the events when there is no access to dexmedetomidine, or if there is restriction in using it. Among advantages of clonidine, one may note that it does not elevate ICP pressure and controls pain, especially in narcotic drug abusers (158). Since clonidine reduces blood pressure and heartbeat, these 2 factors should be monitored in patients receiving this medication. Another advantage of dexmedetomidine over clonidine is that high blood pressure and recurring tachycardia are not of concern 48 hours after discontinuation of dexmedetomidine. 

**Acetylcholinesterase inhibitors**

The anticholinergic mechanism playa roles in pathogenesis of many medications that create delirium. In addition, anticholinergic mechanism plays a role in delirium caused by hypoxia, hypoglycemia, thiamine deficiency, brain trauma damages, and brain strokes as well. Based on this theory, that researchers tested medications that could increase cholinergic system activities (acetylcholinesterase inhibitors) in different studies. However, the results obtained in those studies showed using those medications increased mortality rate in delirium patients; thus, currently, those medications are no longer used in treating delirium. 

## Conclusion

This narrative review was done to provide an overview of delirium definition, diagnosis and its pharmacological management. This review has cited the names of most common medications used in the treatment of delirium. Moreover, in addition to reasons the advantages and disadvantages of the use of those medications have also been discussed. The authors have focused to categorize patients based on the etiology of delirium and to identify the appropriate treatment based on this category to achieve appropriate individualized and purposeful treatment. 
